# Natural Variation in the Control of Flowering and Shoot Architecture in Diploid *Fragaria* Species

**DOI:** 10.3389/fpls.2022.832795

**Published:** 2022-02-24

**Authors:** Guangxun Fan, Javier Andrés, Klaus Olbricht, Elli Koskela, Timo Hytönen

**Affiliations:** ^1^Department of Agricultural Sciences, Viikki Plant Science Center, University of Helsinki, Helsinki, Finland; ^2^Thaer-Institute for Agricultural and Horticultural Sciences, Humboldt-Universität zu Berlin, Berlin, Germany; ^3^Department of Genetics, Genomics and Breeding, NIAB EMR, Kent, United Kingdom

**Keywords:** *Fragaria*, flowering, axillary bud, temperature, photoperiod, *TERMINAL FLOWER1*, *GA20ox4*, Rosaceae

## Abstract

In perennial fruit and berry crops of the Rosaceae family, flower initiation occurs in late summer or autumn after downregulation of a strong repressor *TERMINAL FLOWER1* (*TFL1*), and flowering and fruiting takes place the following growing season. Rosaceous fruit trees typically form two types of axillary shoots, short flower-bearing shoots called spurs and long shoots that are, respectively, analogous to branch crowns and stolons in strawberry. However, regulation of flowering and shoot architecture differs between species, and environmental and endogenous controlling mechanisms have just started to emerge. In woodland strawberry (*Fragaria vesca* L.), long days maintain vegetative meristems and promote stolon formation by activating *TFL1* and *GIBBERELLIN 20-OXIDASE4* (*GA20ox4*), respectively, while silencing of these factors by short days and cool temperatures induces flowering and branch crown formation. We characterized flowering responses of 14 accessions of seven diploid *Fragaria* species native to diverse habitats in the northern hemisphere and selected two species with contrasting environmental responses, *Fragaria bucharica* Losinsk. and *Fragaria nilgerrensis* Schlecht. ex J. Gay for detailed studies together with *Fragaria vesca*. Similar to *F. vesca*, short days at 18°C promoted flowering in *F. bucharica*, and the species was induced to flower regardless of photoperiod at 11°C after silencing of *TFL1*. *F. nilgerrensis* maintained higher *TFL1* expression level and likely required cooler temperatures or longer exposure to inductive treatments to flower. We also found that high expression of *GA20ox4* was associated with stolon formation in all three species, and its downregulation by short days and cool temperature coincided with branch crown formation in *F. vesca* and *F. nilgerrensis*, although the latter did not flower. *F. bucharica*, in contrast, rarely formed branch crowns, regardless of flowering or *GA20ox4* expression level. Our findings highlighted diploid *Fragaria* species as rich sources of genetic variation controlling flowering and plant architecture, with potential applications in breeding of Rosaceous crops.

## Introduction

The Rosaceae family contains economically important perennial crops, ranging from herbaceous species, such as strawberries (*Fragaria* spp.) to fruit trees like apples (*Malus × domestica* Borkh.) or peaches [*Prunus persica* (L.) Batsch; Kurokura et al., 2013]. In strawberries and Rosaceous fruit trees, floral induction takes place during summer or autumn, and flower initials continue developing until late autumn. As the season advances toward winter, these species gradually enter a period of dormancy that is broken during winter after a genetically determined period of cold temperatures called chilling requirement. As the growing season begins in the spring, vegetative growth resumes and blooming occurs (Wilkie et al., 2008; Bangerth, 2009; Kurokura et al., 2013; Costes et al., 2014). Similarities in the seasonal growth cycles of strawberries and Rosaceous fruit trees suggest that species, such as woodland strawberry (*F. vesca* L.) and other diploid strawberries, may be successfully used as models for studying developmental events during the seasonal cycle.

The cues required for floral induction and subsequent floral initiation differ from species to species and may depend on environmental, developmental, agricultural, or genetic factors. For instance, in apple and sweet cherry (*Prunus avium* L.), floral initiation depends on temperature, with species-specific optima (Sønsteby and Heide, 2019; Heide et al., 2020). In the perennial herbaceous model species *F. vesca*, floral induction is highly dependent on the interaction of temperature and photoperiod, and natural populations exhibit differences in their responses to these environmental cues. In some populations, cool temperature of 9°C is sufficient to induce flowering independently of photoperiod, whereas in other populations grown at cool temperature, the promoting effect of short days (SD) is evident (Heide and Sønsteby, 2007). Moreover, a population sampled from the North of Norway shows a strikingly altered yearly growth cycle with an obligatory requirement for vernalization (Heide and Sønsteby, 2007; Koskela et al., 2017). Given that such variation in environmental responses exists within a single species, it is imaginable that extending these studies to other related diploid strawberry species could reveal further adaptations to local environments. Characterizing the available diversity within the *Fragaria* genus may prove useful not only for researchers but also for breeders looking for novel breeding targets to improve climatic adaptation of Rosaceous crops.

As developmental events depend on the timing of meristem differentiation and the meristematic fate itself, studies on meristem fate may provide insights into climatic adaptation of plants. In Rosaceous species, shoot apical meristems (SAMs) located at shoot tips can either generate new vegetative tissues, develop terminal inflorescences, or in some species abort spontaneously (Costes et al., 2014). Meristems located in leaf axils develop axillary buds (AXBs), which can remain latent, develop into vigorously growing long shoots (called stolons in strawberries) or into short shoots (also known as spurs or dwarf shoots in fruit trees and branch crowns in strawberries) with limited extension growth and a rosette-like appearance. In many Rosaceous species strawberries included, terminal meristems borne on short shoots are more prone to receiving the floral induction stimulus and initiating flowers than meristems borne on vigorously growing long shoots (Hytönen et al., 2004; Heide and Sønsteby, 2007; Wilkie et al., 2008; Sønsteby and Heide, 2019). Therefore, the balance between short and long shoots defines the yield potential and affects the choice and expenses of cultural practices, such as pruning or training.

Molecular studies in *F. vesca* (Koskela et al., 2012; Rantanen et al., 2015) and cultivated strawberry *Fragaria* × *ananassa* Duch. (Koskela et al., 2016) have highlighted the role of *TERMINAL FLOWER1* (*TFL1*) as a floral repressor. In *F. vesca*, photoperiodic pathway culminates in the regulation of *SUPPRESSOR OF OVEREXPRESSION OF CONSTANS1* (*FvSOC1*), which activates *FvTFL1* in long days (LDs), and flower induction occurs in SDs after gradual downregulation of *FvSOC1* and *FvTFL1* (Koskela et al., 2012; Mouhu et al., 2013; Rantanen et al., 2015; Kurokura et al., 2017). However, this photoperiodic pathway regulates flowering only within a narrow temperature range between 13 and 20°C (Rantanen et al., 2015). Lower temperatures repress *FvTFL1* and induce flowering independently of photoperiod, while higher temperatures activate *FvTFL1* and inhibit flowering regardless of the photoperiod (Rantanen et al., 2015).

The function of *TFL1* in Rosaceous species is conserved. Silencing or knocking out *TFL1* homologs in apple and pear (*Pyrus communis* L.) result in reduced juvenility, precocious flowering, and even perpetual flowering (Kotoda et al., 2006; Flachowsky et al., 2012; Freiman et al., 2012; Charrier et al., 2019). Likewise, loss of function of *TFL1* homologs in roses (*Rosa* spp.) and *F. vesca* lead to perpetual flowering (Iwata et al., 2012; Koskela et al., 2012; Bai et al., 2021). The seasonal expression pattern of *TFL1* is highly connected to the yearly growth cycle in apple, *F. vesca*, and roses, as *TFL1* is activated in the SAM during the vegetative growth phase and downregulated before the floral induction to allow flower initiation (Mimida et al., 2011; Iwata et al., 2012; Kurokura et al., 2013; Koskela et al., 2017). With such a conserved function and expression patterns across rosaceous species, it is reasonable to expect that results from studies on *TFL1* in one species are applicable to other species.

Regulation of AXB fate in Rosaceae has been mainly studied at the phenotype level, perhaps because the molecular processes taking place within the well-protected AXB are difficult to examine. However, recent reports in *F. vesca* demonstrated that stolon development requires *GIBBERELLIN 20-OXIDASE4* (*FvGA20ox4*) that is activated within the AXBs under LD conditions *via* an *FvSOC1*-dependent photoperiodic pathway at 18°C (Mouhu et al., 2013; Tenreira et al., 2017; Andrés et al., 2021). Higher temperature of 22°C upregulates *FvGA20ox4* independently of *FvSOC1*, whereas at cooler temperature (11°C), *FvGA20ox4* is de-activated in both SD and LD conditions (Andrés et al., 2021). Cool temperatures, as well as SDs at 18°C, promote BC development instead of stolons in the seasonal flowering *F. vesca*, and although the same environmental cues induce flowering, these two processes can occur independently (Andrés et al., 2021). However, the fate of the youngest AXB located immediately below the SAM is directly dependent on the vegetative/generative status of the SAM; if the SAM is induced to flower, the youngest AXB develops a branch crown to continue the growth of the plant in a sympodial fashion (Sugiyama et al., 2004; Andrés et al., 2021).

Studying phenotypic and genetic variation present in related species is based on the rationale that species sampled from diverse environments have faced different selection pressures, leading to evolution of local adaptation and phenotypical differences. We took advantage of this rationale to study environmental responses in wild diploid species of *Fragaria* originating from a wide geographical range. Our collection of wild diploid *Fragaria* included accessions native to high altitude (*F. bucharica* and *F. nubicola* Lindl. from the Himalayas) as well as accessions endemic to less harsh environments (*F. iinumae* Makino growing in the Japanese archipelago and *F. nilgerrensis* and *F. pentaphylla* Losinsk. from South East Asia). In addition, we included our Finnish reference accession of *F. vesca* that is adapted to temperate climate, *F. viridis* Weston from Central Europe and *F. chinensis* Losinsk. from North Western China. We analyzed the collection in terms of flowering habits and vegetative development and selected *F. bucharica* and *F. nilgerrensis* for detailed analysis together with *F. vesca*. Our results indicated that *TFL1* homologs are key integrators of temperature and photoperiodic cues in these three species and that altered regulation of *TFL1* may explain variation in their flowering habits. We also found that the activities of *GA20ox4* homologs correlated with AXB fate in the studied species.

## Materials and Methods

### Plant Material

Fourteen accessions of seven wild diploid strawberry species (Supplementary Table 1), provided by the Professor Staudt Collection maintained by Hansabred GmbH Co. KG in Dresden, Germany, were included in initial screening of flowering responses. If there is any interest on the plant material, the corresponding author or Hansabred^1^ should be contacted. Other experiments included *F. nilgerrensis* accession #1, *F. bucharica* accession #1, and seasonal flowering *F. vesca* accession FIN56 (PI551792, National Clonal Germplasm Repository, Corvallis, OR, United States) as a control. All plant materials used in this study were propagated from stolon cuttings in a greenhouse. Plants were first grown on jiffy pellets (Jiffy Products International) for 3 weeks and then transplanted to 8 cm × 8 cm pots with fertilized peat (Kekkilä, Finland). Liquid fertilizer (Kekkilä, N-P-K: 17-4-25, Finland) was given to the plants biweekly.

### Treatments and Observations

Plants were grown in a greenhouse under 18 h LD at 18°C for 4 weeks before the experiments started. In the greenhouse, plants were illuminated by natural light, and high-pressure sodium lamps (Airam 400 W, Kerava, Finland) at a photosynthetic photon flux density (PPFD) of 120 μmol m^−2^ s^−1^ were used to extend the day length. Temperature (11 and 18°C) and photoperiod (12-h SD and 18-h LD) treatments were carried out in growth chambers equipped with LED lamps (AP67, Valoya, Finland; 200 μmol m^−2^ s^−1^ of PPFD). The experimental details are described in the figure legends. During the experiments, the number of leaves, stolons, and BCs were observed weekly, and stolons were removed after recording. For flowering time observations, both the numbers of leaves from the primary leaf rosette and the number of days when the first fully open flower emerged were recorded. In this study, the BC number referred to the number of axillary leaf rosettes excluding the sympodial BC arising from the topmost axil upon floral initiation.

### Gene Expression Data

Shoot apex samples were collected for gene expression analysis. Total RNA was extracted as described by Koskela et al. (2012) and treated with rDNase (Macherey-Nagel GmbH, Düren, Germany) according to the manufacturer’s instructions. cDNA was synthesized from 500 ng of total RNA using ProtoScript II Reverse Transcriptase according to manufacturer’s instructions (New England Biolabs). SYBR Green I master mix was used for quantitative real-time PCR (qRT-PCR) in a total reaction volume of 10 μl and analyzed by LightCycler 480 instrument (Roche) as described by Koskela et al. (2012). Four biological replicates and three technical replicates were used for qRT-PCR analysis using the primers listed in Supplementary Table 2. Relative expression levels were calculated by ΔΔCt method as described by Pfaffl (2007). *FvMSI1* (*MULTICOPY SUPPRESSOR OF IRA1*) was used as a reference gene for normalization.

### Statistical Analyses

Either logistic regression or ANOVA was conducted to test the main factors, and pairwise comparisons were performed by Tukey HSD. The statistical analyses were done using R.4.1.0 (R Core Team, 2021), the stats (v4.1.0; R Core Team, 2021), and the DescTools (v0.99.42; Andri et al., 2021) packages.

## Results

### Flowering Time Variation Among Diploid Strawberry Species

**Figure 1:**
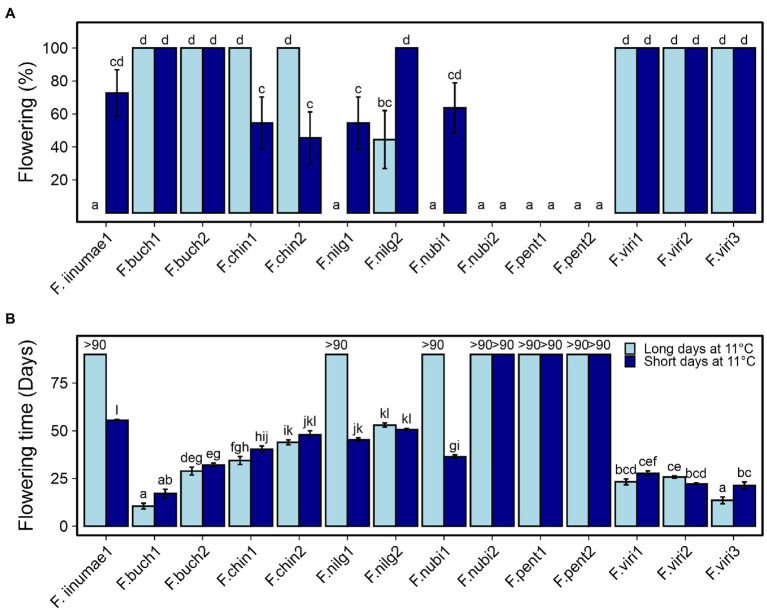
Flowering responses of diploid species to 12 h short day (SD) and 18 h long day (LD) treatments at 11°C. Percentage of flowering plants **(A)** and days to the first open flower **(B)**. Stolon-propagated plants were grown under 18- or 12-h photoperiod at 11°C for 6 weeks followed by LDs (18-h) at 18°C. Flowering was recorded every other day, starting after the treatments. Error bars represent the SEM (*n* = 7–11), and different letters indicate significant differences calculated by ANOVA and Tukey’s test (*p* < 0.05). For accessions that remained vegetative, the number of days recorded is shown (>90).

To get the first insights into flowering responses to environmental cues in different diploid strawberry species, 14 accessions of seven species were subjected to 18 h LD and 12 h SD treatments at 11°C for 6 weeks, followed by flowering observations in LDs at 18°C. Eleven accessions showed clear flowering response to at least one of the treatments (Figure 1A). All the plants of two *F. bucharica* and three *F. viridis* accessions flowered after both LD and SD treatments, showing a similar photoperiod-independent flowering response to cool temperature as SD genotypes of *F. vesca* (Heide and Sønsteby, 2007; Rantanen et al., 2015). In the two *F. chinensis* accessions, floral induction took place in all the LD-grown plants, whereas only roughly half of the plants were induced under SD conditions. Opposite photoperiodic response was found in two accessions of *F. nilgerrensis*, in which SDs resulted in a higher percentage of flowering plants. In *F. nilgerrensis* #1, as well as in *F. iinumae* #1 and *F. nubicola* #1, flowering occurred only after the SD treatment (Figure 1A). Finally, flowering was not observed in *F. nubicola* #2 and the two *F. pentaphylla* accessions under either photoperiod. There were also significant differences in flowering time between the species and accessions (Figure 1B). Accessions of *F. bucharica* and *F. viridis* flowered rapidly after the temperature treatment independently of photoperiod, except for *F*. *viridis* #3 in which LDs promoted flowering, while *F. iinumae* and *F. nilgerrensis* accessions flowered significantly later.

Because we were not able to induce *F. nubicola* #2 and the two *F. pentaphylla* accessions to flower under SDs or LDs at 11°C, we tested if a prolonged cold treatment could induce them to flower. After 1 months in 12 h SD at 14°C–15°C, plants were moved to 5°C–6°C for about 4 months. This treatment induced flowering in all the plants of both *F. pentaphylla* accessions that did not flower after 11°C treatment (Supplementary Table 3). However, *F. nubicola* #2 did not flower, and inductive conditions for this accession remained an enigma. Furthermore, *F. iinumae* #1 did not flower, although it flowered in a previous experiment. In conclusion, the screening experiment with 14 *Fragaria* accessions revealed an interesting diversity of flowering responses.

### The Effect of Photoperiod on Flowering Time and AXB Fate at 18°C

Next, we studied flowering responses of two accessions in more detail and compared them with the seasonal flowering *F. vesca* reference genotype, FIN56. We selected *F. bucharica* #1 because it flowered first in the initial screening experiment and *F. nilgerrensis* #1 because it flowered late and had a clear photoperiodic response at 11°C (Figure 1; the rest of the experiments included only one genotype per accession and therefore *F. bucharica* #1 and *F. nilgerrensis* #1 are referred to as *F. bucharica* and *F. nilgerrensis* from here onward). First, we decided to subject the three species to LDs or SDs at 18°C, because the phenotypical responses and gene expression profiles in *F. vesca* are well characterized under these conditions.

**Figure 2:**
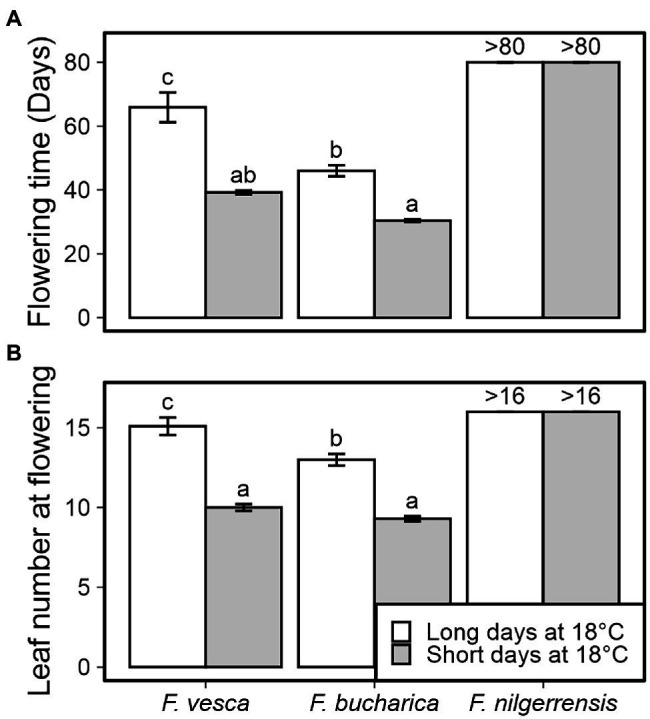
Flowering time in three diploid strawberry species grown in SDs or LDs at 18°C. Number of days **(A)** and number of leaves developed **(B)** until the first flower opened. Stolon-propagated plants were grown in LDs (18-h) or SDs (12-h) at 18°C for 6 weeks followed by LDs (18-h) at 18°C. Flowering time was recorded every other day and leaf number was scored at flowering time. Error bars represent the SEM (*n* = 10), and different letters indicate significant differences calculated by ANOVA and Tukey’s test (*p* < 0.05). For plants that remained vegetative, the number of days recorded (>80) or the number of leaves observed (>16) is shown.

A 6-week SD treatment at 18°C induced early flowering in both *F. vesca* and *F. bucharica* (Figure 2). However, also LD-grown control plants of both species flowered, but significantly later than SD-grown plants, and longer delay was observed in *F. vesca*. In contrast, *F. nilgerrensis* did not flower under either photoperiod at 18°C.

**Figure 3:**
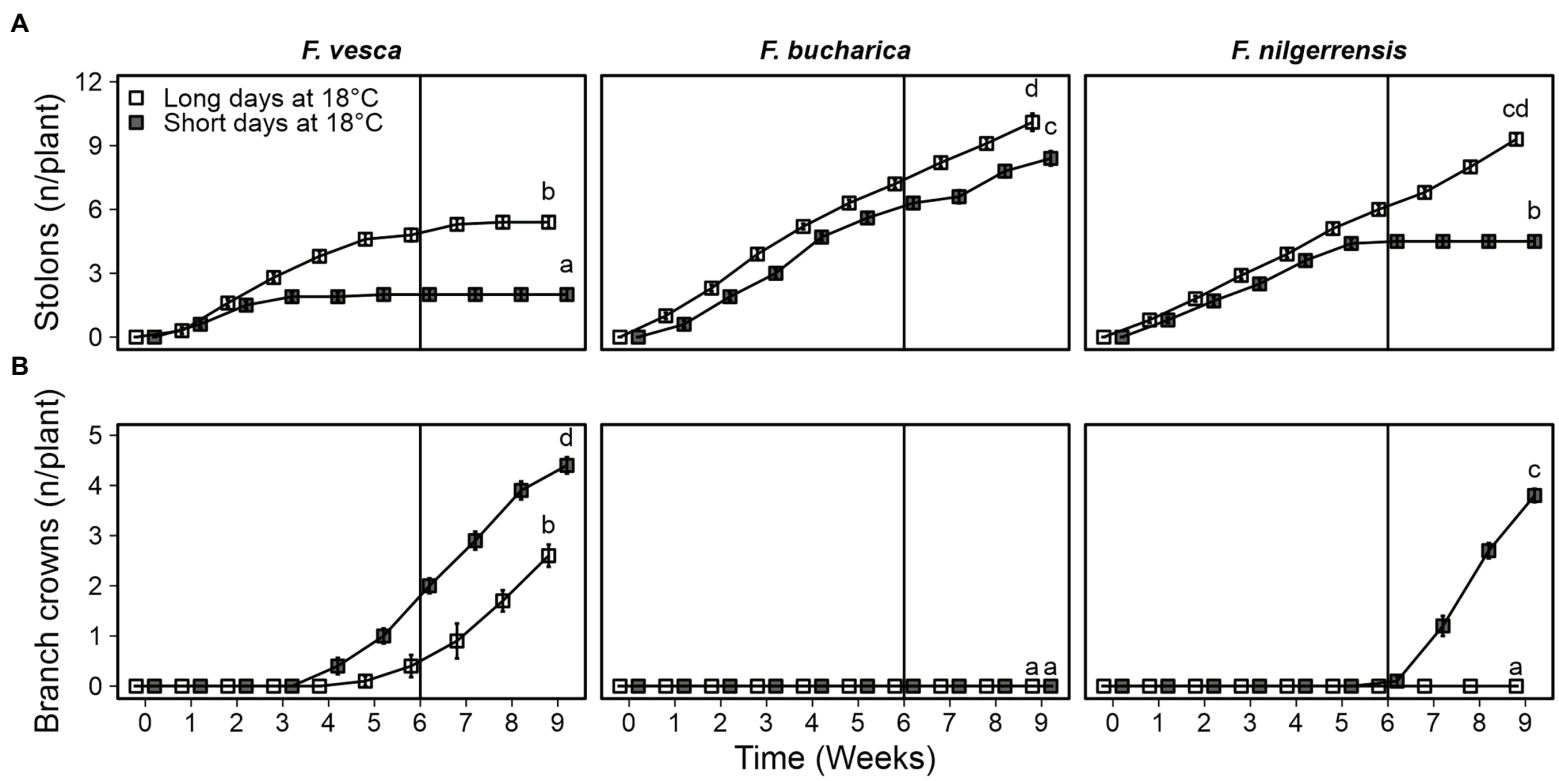
Axillary bud fate of three diploid strawberry species grown in SDs or LDs at 18°C. Number of stolons **(A)** and branch crowns **(B)** per plant. Stolon-propagated plants were grown in LDs (18-h) or SDs (12-h) at 18°C for 6 weeks followed by LDs (18-h) at 18°C. Number of stolons and branch crowns was recorded weekly until week 9. Error bars represent the SEM (*n* = 10) and different letters indicate significant differences calculated by ANOVA and Tukey’s test (*p* < 0.05).

*Fragaria vesca* ceased stolon development after 3 weeks of SDs. The final number of stolons in *F. vesca* at the end of the experiment was significantly higher in LDs than in SDs (Figure 3A). *Fragaria bucharica* did not stop stolon development under either photoperiod, although LDs slightly promoted stolon development also in this species (Figure 3A). Stolon development in *F. nilgerrensis* ceased after 5 weeks in SDs at 18°C, whereas LDs promoted stolon development until the end of the experiment (Figure 3A).

The three species behaved very differently in terms of BC development at 18°C. In *F. vesca*, BC development was observed in both photoperiods, with SDs promoting BC development (Figure 3B). Intriguingly, *F. bucharica* did not develop any BCs under either photoperiod. In *F. nilgerrensis*, 6 weeks of SDs at 18°C strongly promoted BC development whereas the LD-grown *F. nilgerrensis* did not develop any BCs (Figure 3B). Taken together, all species responded to photoperiod, but showed contrasting responses; in *F. vesca*, photoperiod affected both flowering and AXB fate, *F. bucharica* showed SD promotion of flowering but no strong photoperiodic effects on AXB fate, while in *F. nilgerrensis*, photoperiod affected only AXB fate.

### Altered Expression of Key Genes Is Associated With Different Phenotypical Responses at 18°C

**Figure 4:**
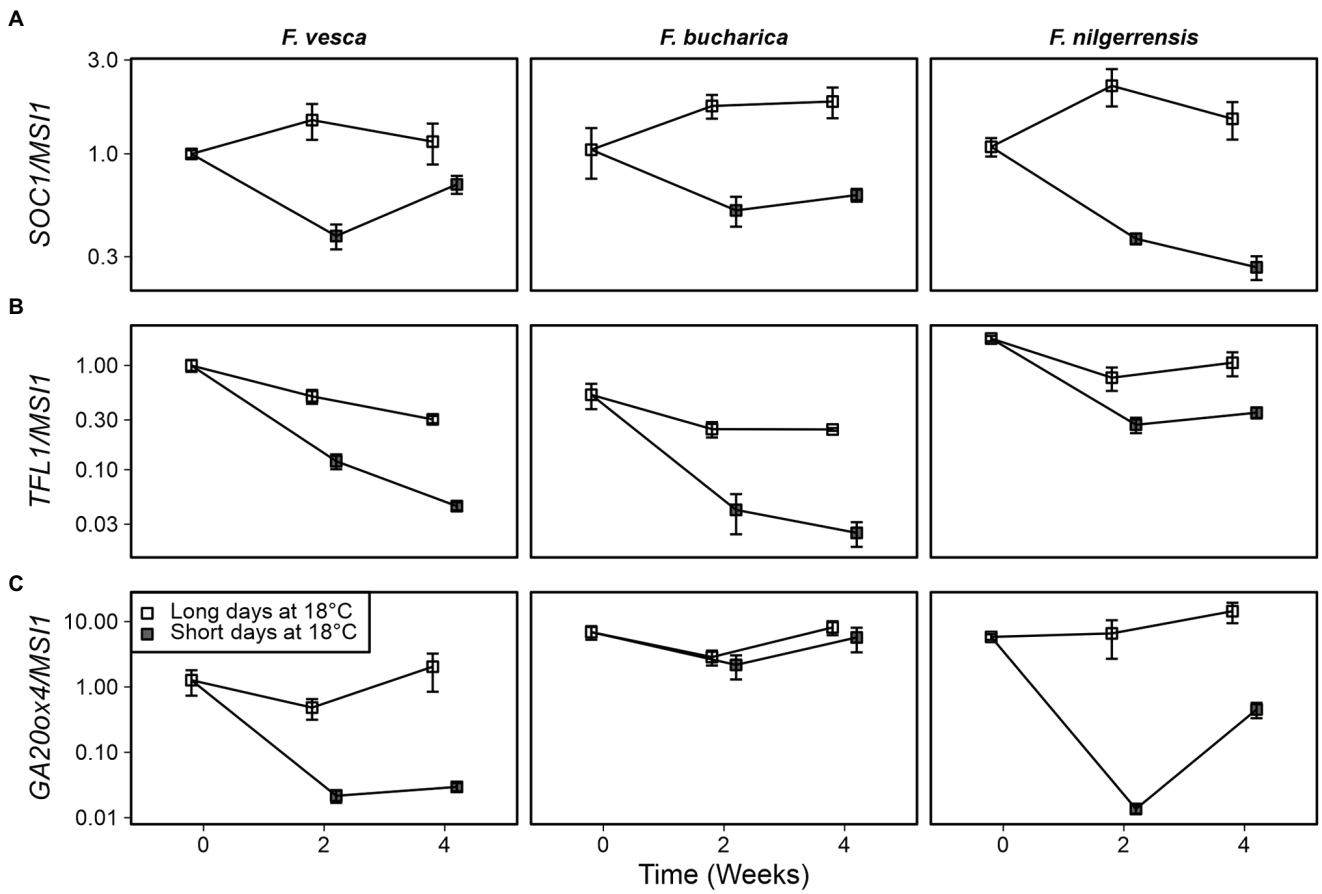
Gene expression patterns in three diploid strawberry species grown under SDs or LDs at 18°C. *SUPPRESSOR OF OVEREXPRESSION OF CONSTANS1* (*SOC1*) **(A)**, *TERMINAL FLOWER1* (*TFL1*) **(B)**, and *GIBBERELLIN 20-OXIDASE4* (GA20ox4) expression **(C)** in shoot apical samples is shown. Stolon-propagated plants were grown in LDs (18-h) or SDs (12-h) at 18°C, and shoot apical samples were collected at the beginning of the treatments, and 2 and 4 weeks later. Week 0, *Fragaria vesca* samples were used as a calibrator for relative expression analysis. Error bars represent the SEM (*n* = 3–4).

To gain an initial idea of how the photoperiodic pathway functions in *F. bucharica* and *F. nilgerrensis*, we decided to study the expression of *SOC1*, *TFL1*, and *GA20ox4* in these species in comparison to *F. vesca*. The *SOC1* genes had a very clear photoperiodic response in all three species and the expression patterns were very similar. *SOC1* remained active in LDs at 18°C, whereas SDs downregulated the gene (Figure 4A), suggesting that the photoperiodic regulation of *SOC1* at 18°C is conserved in these diploid species.

Earlier experiments in *F. vesca* have shown that *FvTFL1* is gradually de-activated after transferring the plants to SD conditions (Koskela et al., 2012). In our current experiment, *TFL1* expression dropped to low levels in both *F. vesca* and *F. bucharica* within 2 weeks under SD conditions, and the expression further declined until week 4 (Figure 4B). In *F. nilgerrensis*, *TFL1* expression in SDs remained at higher level than in the other two species, although there was still a clear photoperiodic effect, indicating that the high activity of *TFL1* may inhibit floral induction in the experimental conditions used. Moreover, regulation of *TFL1* in *F. nilgerrensis* (*FnTFL1*) does not follow the expression pattern of *SOC1*, implying that *FnTFL1* is regulated by factor(s) other than the *SOC1*-dependent pathway. The analysis of available *F. vesca* and *F. nilgerrensis* genomic sequences showed extensive variation in putative regulatory regions of *TFL1* (Supplementary Figure 1; Edger et al., 2017; Zhang et al., 2020), which may explain differential regulation of *TFL1* in these species.

*GIBBERELLIN 20-OXIDASE4* was rapidly downregulated upon exposure to SDs in both *F. vesca* and *F. nilgerrensis* (Figure 4C). Concurring with the stolon phenotype, we found that the expression of *GA20ox4* in *F. bucharica* remained at a high level in LDs and SDs at 18°C. The finding in *F. bucharica* suggests that in this species the regulation of *GA20ox4* is uncoupled from the expression of *SOC1*, leading to continuous and photoperiod-independent development of stolons at 18°C.

### Three Diploid *Fragaria* Species Exhibit Distinct Phenotypic Responses at 11°C

**Figure 5:**
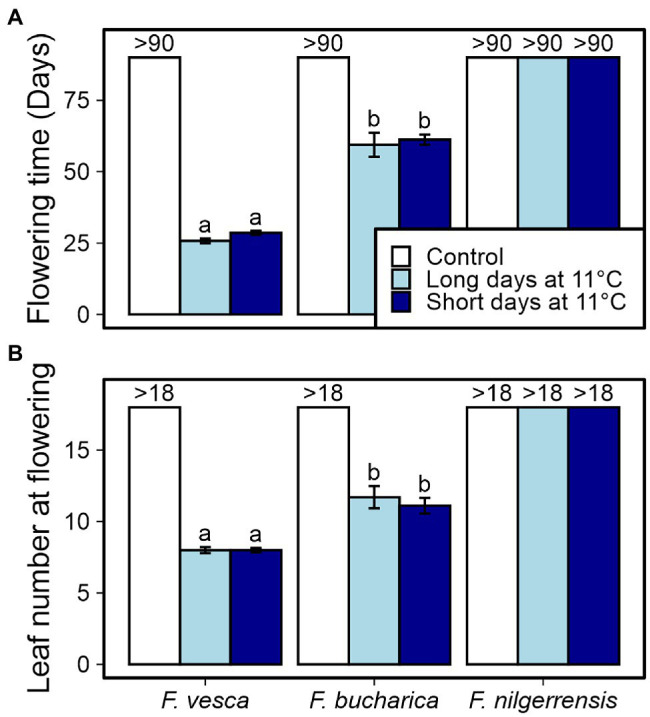
Flowering characterization of three diploid strawberry species grown under SDs or LDs at 11°C. Number of days **(A)** and number of leaves developed **(B)** until the first flower opened. Stolon-propagated plants were grown in LDs (18-h) or SDs (12-h) at 11°C for 6 weeks followed by LDs (18-h) at 18°C. Control plants were grown in LDs (18-h) at 18°C from the beginning of the experiment. Flowering time was recorded every other day, starting after the treatments. Error bars represent the SEM (*n* = 10) and different letters indicate significant differences calculated by ANOVA and Tukey’s test (*p* < 0.05). For plants that remained vegetative, the number of days recorded (>90) or the number of leaves observed (>18) is shown.

Next, we wanted to study the phenotypical responses of the three *Fragaria* species at 11°C at a more detailed level. We repeated the photoperiodic experiment at 11°C and observed flowering and AXB fates. It has been earlier shown in *F. vesca* that cool temperature induces flowering independently of photoperiod (Heide and Sønsteby, 2007; Rantanen et al., 2015; Andrés et al., 2021). We found similar photoperiod-independent response in both *F. vesca* and *F. bucharica* after 6 weeks at 11°C with significantly later flowering in *F. bucharica*, while the control plants grown under LD conditions at 18°C remained vegetative until the end of the experiment (Figure 5). By contrast, no flowering plants were observed in *F. nilgerrensis* in this experiment, and only 50% of plants flowered in the initial screening under SDs at 11°C, indicating that a 6-week treatment at 11°C is not sufficient for complete floral induction in this species.

**Figure 6:**
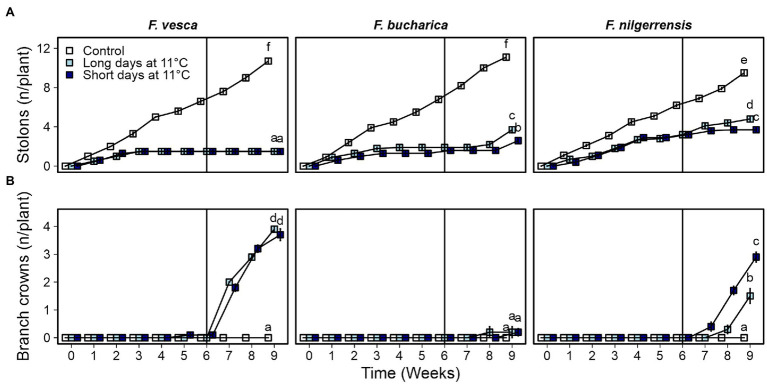
Axillary bud fate in three diploid strawberry species grown under LDs or SDs at 11°C. Number of stolons **(A)** and branch crowns **(B)** per plant. Stolon-propagated plants were grown under LDs (18-h) or SDs (12-h) at 11°C for 6 weeks and subsequently in LDs (18-h) at 18°C Control plants were grown continuously under LDs (18-h) at 18°C. Number of stolons and branch crowns were recorded weekly until week 9. Error bars represent SEM (*n* = 10) and different letters indicate significant differences calculated by ANOVA and Tukey’s test (*p* < 0.05).

We found differences in the environmental regulation on AXB fate among the three diploid strawberry species. Overall, in all species, the control plants developed stolons continuously at a relatively stable speed throughout the experiment (Figure 6A). Cool temperature strongly suppressed stolon development in *F. vesca* and stolon development did not resume after returning the plants to 18°C. Similarly to *F. vesca*, stolon development ceased in *F. bucharica* after 3 weeks at 11°C under both LD and SD conditions, but in contrast to *F. vesca*, *F. bucharica* started stolon development again 2 weeks after the treatments. In *F. nilgerrensis*, stolon development was markedly slowed down at cool temperature compared with the control plants, but no clear cessation of stolon development was observed. On the other hand, *F. bucharica* and *F. nilgerrensis* plants subjected to LDs at 11°C had significantly more stolons on week 9 than plants grown in SDs. In *F. vesca*, such a photoperiodic effect was not observed.

The three *Fragaria* species differed in terms of branch crown development. *Fragaria vesca* started to develop BCs independently of the photoperiod immediately after the 6-week period at 11°C (Figure 6B). On the contrary, *F. bucharica* developed very few BCs after the photoperiodic treatments at 11°C, although it behaved similarly to *F. vesca* in terms of stolon development and all the plants flowered. *F. nilgerrensis* started to develop BCs after the photoperiodic treatments at 11°C with SDs promoting BC development (Figure 6B). It is noteworthy that BC development in *F. nilgerrensis* occurred independent of floral induction, as none of the plants flowered. The control plants grown continuously under LDs at 18°C did not develop BCs in these species. Taken together, our data indicate that AXB fate in the three studied *Fragaria* species is controlled by different mechanisms; in *F. vesca*, cool temperature of 11°C promotes BC development and suppresses stolon development independently of photoperiod, while AXB fate in *F. nilgerrensis* is clearly dependent on photoperiod. Moreover, BC development in *F. bucharica* appears to be endogenously regulated, as neither photoperiod nor temperature affected BC development.

### The Expression of Key Genes Correlates With Flowering and AXB Fate at 11°C

**Figure 7:**
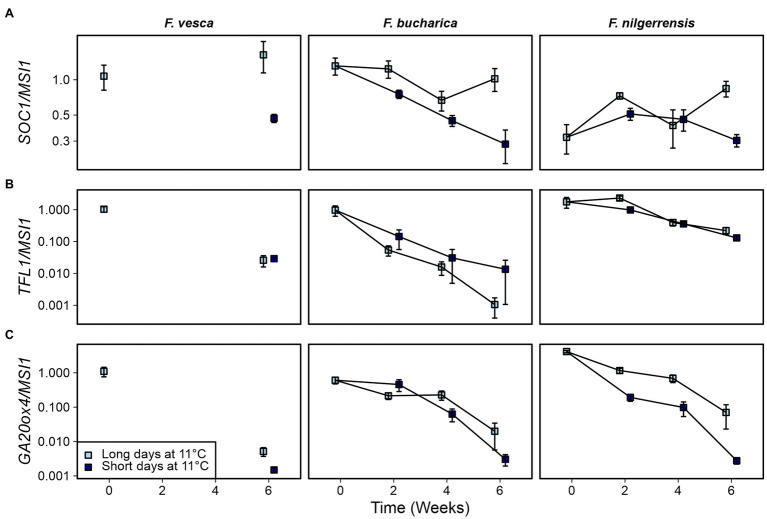
Gene expression patterns in three diploid strawberry species grown in SDs or LDs at 11°C. *SOC1* **(A)**, *TFL1* **(B)**, and *GA20ox4* expression **(C)** in shoot apical samples. Stolon-propagated plants were grown in LDs (18-h) or SDs (12-h) at 11°C, and shoot apical samples were collected at the beginning of the treatments, and 2, 4, and 6 weeks later. Week 0, *Fragaria vesca* samples were used as calibrator for relative expression analysis. Error bars represent the SEM (*n* = 3–4).

Next, we wanted to examine whether the phenotypical differences observed in the three *Fragaria* species at 11°C could be explained by altered expression of key genes. Earlier studies in *F. vesca* suggest that *FvSOC1* is activated by LDs at 10°C, albeit to a lesser extent than at higher temperatures (Rantanen et al., 2015). In our current experiments at 11°C, we saw clear SD-dependent downregulation of *SOC1* in *F. vesca*, as well as in *F. bucharica* (Figure 7A; Supplementary Figure 2). In *F. nilgerrensis*, the pattern of *SOC1* expression was not very clear, although by week 6 the level of *SOC1* mRNA was lower in SDs than in LDs.

As shown earlier by Rantanen et al. (2015), *FvTFL1* in *F. vesca* is de-activated independently of photoperiod at 11°C. *TFL1* was gradually downregulated at 11°C also in our current experiments in both *F. vesca* and *F. bucharica*, and *F. bucharica* showed stronger downregulation of *TFL1* than *F. vesca* (Figure 7B; Supplementary Figure 2). On the contrary, downregulation of *TFL1* in *F. nilgerrensis* occurred slower, and at week 6, *TFL1* expression was still higher in *F. nilgerrensis* than in the other species.

We also analyzed the expression of *GA20ox4*. The effect of 11°C treatment on *GA20ox4* activity was very clear for all the species. In *F. vesca* and *F. bucharica*, the expression level was much lower after 6 weeks at 11°C than in the beginning of the experiment, correlating with the lack of stolon development in these two species (Figure 7C). Results in these species also showed that this downregulation occurred gradually in both photoperiods (Figure 7C; Supplementary Figure 2). By contrast, although *GA20ox4* expression in *F. nilgerrensis* also declined gradually, it was clearly more downregulated in SDs than in LDs from week 2 onward. The difference between photoperiods was even more evident by week 6, being in line with the effect of photoperiod on stolon development in this species.

## Discussion

The molecular mechanisms regulating floral induction and AXB fate as a response to environmental conditions are starting to emerge in the diploid model species *F. vesca* (Hytönen and Kurokura, 2020; Andrés et al., 2021). To gain a broader view on the diversity of regulation of flowering and AXB fate within the *Fragaria* genus, we studied these responses in a panel of wild diploid strawberry species with diverse geographical origins ranging from Asia to Europe. Some of these species inhabit very local habitats while some are widely spread around the Northern Hemisphere (Liston et al., 2014). Here, we characterized the flowering habits of 14 accessions from seven wild diploid strawberry species under controlled environmental conditions. Based on their diverse flowering responses, we further selected two representative species, *F. bucharica* and *F. nilgerrensis*, and analyzed their flowering habits, AXB fate, and expression of key genes related to these biological processes in comparison to the reference model species *F. vesca*.

### Diversity of Flowering Responses in Diploid *Fragaria* Species

We discovered a diversity of flowering responses in our collection of diploid *Fragaria* species. *F. bucharica* and *F. viridis* were clearly photoperiod-insensitive at cool temperature and flowered rapidly after both SD and LD treatments at 11°C, similarly to the model species *F. vesca* (Figure 1; Rantanen et al., 2015). These results were in line with the earlier field observations on facile floral induction in these species; *F. bucharica* flowered twice during the growing season when grown in Germany (Staudt, 2006), and *F. viridis* was described as “remontant” under field conditions in South East England (Sargent et al., 2004), as also observed in the Professor Staudt Collection in Germany (data not shown). In contrast to these species, the promoting effect of SDs was obvious in *F. iinumae*, *F. nilgerrensis*, and *F. nubicola* #1, while LDs advanced flowering in *F. chinensis* at 11°C, a response that has not been previously described in *Fragaria* (Figure 1). Finally, *F. pentaphylla* and *F. nubicola* #2 did not flower at all after 11°C treatments. The diverse responses observed in our collection warrant further investigation to uncover mechanisms controlling flowering time variation in these species.

In general, diploid strawberries flower in their original habitats in spring/summer after overwintering (Staudt et al., 2003; Staudt, 2005, 2006, 2009), and all tested accessions of *F. iinumae*, *F. nilgerrensis*, *F. nipponica*, *F. nubicola*, *F. pentaphylla*, *F. viridis*, and *F. vesca* flowered and produced fruits after overwintering in the field in South East England (Sargent et al., 2004). Likewise, a long-term exposure to 5°C–6°C in a greenhouse without supplemental light promoted flowering in the majority of the accessions we tested, excluding *F. iinumae* and *F. nubicola* #2 (Supplementary Table 3). In contrast, Bors and Sullivan (2005) reported that a 2-month period at constant −1°C could not induce some accessions of *F. nilgerrensis*, *F. nubicola*, *F. pentaphylla*, and *F. viridis* to flower and an additional floral induction treatment under 10-h SDs at 18/15°C (day/night) was necessary. As the experimental conditions, as well as the plant materials, differ between the studies (Sargent et al., 2004; Bors and Sullivan, 2005), direct comparisons are difficult. The contrasting results highlight the need for experimentation under controlled climate to uncover the exact environmental conditions required for floral induction in *Fragaria* species. Furthermore, observations in the Professor Staudt Collection indicate that plant age should also be considered (data not shown).

### Variation in *TFL1* Regulation Correlates With Contrasting Flowering Habits in Three Diploid *Fragaria* Species

*TERMINAL FLOWER1* is a strong floral repressor in different Rosaceous species (Kotoda et al., 2006; Flachowsky et al., 2012; Freiman et al., 2012; Iwata et al., 2012; Koskela et al., 2012, 2016; Charrier et al., 2019). We found a clear association between *FvTFL1* de-activation and flowering in *F. vesca*, corroborating the earlier findings by Rantanen et al. (2015), and a similar association was found in *F. bucharica*. In both species, SDs suppressed *TFL1* and promoted flowering at 18°C, whereas *TFL1* downregulation and floral induction occurred independently of photoperiod at 11°C (Figures 2, 4B, 5, 7B). However, we were unable to induce flowering in *F. nilgerrensis* in SDs at 18°C, and after 6-week treatments at 11°C, flowering was observed only in some plants in one of the two experiments. As *F. nilgerrensis TFL1* mRNA showed overall higher level, slower downregulation at 11°C, and weak photoperiodic response at 18°C compared with other species, this species may require longer exposure to cool temperatures or photoperiods shorter than 12 h to downregulate *TFL1* below the threshold level for flower induction. In consistence with this hypothesis, the species originates from low latitude area with relatively mild seasonal changes of temperature and photoperiod, where the period of flower-inductive conditions is longer than in the colder habitats of *F. vesca* and *F. bucharica* (Liston et al., 2014). However, while the environmental conditions required for floral induction differ between the species, the role of *TFL1* as a floral repressor appears to be conserved in *F. vesca*, *F. bucharica*, and *F. nilgerrensis*.

Earlier experiments in *F. vesca* identified *FvSOC1* as a major LD-activated promoter of *FvTFL1* expression at 18°C (Mouhu et al., 2013). We observed LD-dependent activation of *SOC1* expression at 18°C for *F. vesca*, *F. bucharica*, and *F. nilgerrensis* (Figure 4A), suggesting that the photoperiodic pathway upstream of *SOC1* functions similarly in the three species. However, the downregulation of *TFL1* expression coincided with that of *SOC1* only in *F. vesca* and *F. bucharica*, as in *F. nilgerrensis*, *FnTFL1* activity remained at a relatively high level under both photoperiods although *FnSOC1* was strongly downregulated by SDs. This indicates an uncoupling of *FnSOC1* and *FnTFL1* expression patterns that is reminiscent of the events taking place in *F. vesca* at 23°C; at this temperature, *FvTFL1* is upregulated by an unknown pathway independently of *FvSOC1* (Rantanen et al., 2015). It is possible that the temperature threshold for the activation of this unidentified pathway is lower in *F. nilgerrensis* than in *F. vesca*, leading to photoperiod- and *FnSOC1*-independent upregulation of *FnTFL1* already at 18°C. Finally, at 11°C, downregulation of *TFL1* was not clearly associated with changes in *SOC1* expression level in any of the three species, as previously reported in *F. vesca* (Rantanen et al., 2015).

In conclusion, *TFL1* is a key integrator of environmental signals in the three studied diploid strawberry species, and variation in the downregulation of *TFL1* may explain the observed differences in their photoperiodic and temperature responses. Further studies are needed to explore what are the molecular mechanisms controlling variation in *TFL1* regulation.

### *GA20ox4* Promotes Stolon Development in the Three *Fragaria* Species

Although the regulation of AXB fate in *F. vesca* has received attention in the recent years (Tenreira et al., 2017; Caruana et al., 2018; Li et al., 2018; Qiu et al., 2019; Andrés et al., 2021), AXB fate regulation has remained unexplored in other wild *Fragaria* species. We found that, in line with previous studies in *F. vesca* (Mouhu et al., 2013); SDs at 18°C completely inhibited stolon development in *F. vesca* and *F. nilgerrensis* after 3 and 6 weeks of treatments, respectively. However, stolon development of *F. bucharica* was reduced only slightly under SDs (Figure 3B). In *F. vesca*, this photoperiodic response is mediated *via FvSOC1* that promotes stolon formation in LDs by upregulating *FvGA20ox4* in AXBs, and the downregulation of these genes stops stolon formation (Andrés et al., 2021). In our current experiment, *GA20ox4* expression was in line with that of *SOC1* in *F. vesca* and *F. nilgerrensis* at 18°C, but not in *F. bucharica* that exhibited clear photoperiodic regulation of *SOC1* but barely noticeable differences in *GA20ox4* expression (Figure 4). Andrés et al. (2021) found that, at 23°C, *FvGA20ox4* expression is promoted by an unknown factor in SD-grown *F. vesca* plants, in spite of downregulation of *FvSOC1*. Perhaps the same factor upregulated *GA20ox4* at 18°C in SD-grown *F. bucharica* plants in the current experiment.

Corroborating with earlier studies in *F. vesca*, stolon development ceased in all three species at 11°C regardless of the photoperiod, but this happened later in *F. nilgerrensis* than in other species (Figure 6A; Heide and Sønsteby, 2007; Andrés et al., 2021). The cessation of stolon development was associated with gradual downregulation of *GA20ox4* expression in all three species indicating that *GA20ox4* controls stolon development also in *F. bucharica* and *F. nilgerrensis* in these conditions. This finding is in line with the result of Andrés et al. (2021), who observed photoperiod-independent downregulation of *FvGA20ox4* in the *F. vesca* accession “Hawaii-4” at cool temperature. It is notable that the expression of *GA20ox4* does not follow the expression pattern of *SOC1* at 11°C, not in our current experiment (Figure 7), nor in the earlier study by Andrés et al. (2021). These data indicate that, at 11°C, *GA20ox4* expression is regulated by factors other than *SOC1* in the three species.

### Different Pathways Regulate Branch Crown Development in the Three Species

Although flower-inductive treatments promoted BC development in *F. vesca*, BC formation did not correlate with flowering in *F. bucharica* and *F. nilgerrensis*, highlighting the diverged regulation of BC development in the three species. In *F. nilgerrensis*, SDs and cool temperature activated BC development independently of flowering, which was previously found also in late- and non-flowering *F. vesca* mutants with high *FvTFL1* expression levels (Andrés et al., 2021). In *F. bucharica*, none of our tested conditions could promote BC development; although we witnessed clear photoperiodic and temperature regulation of flowering (Figures 3B, 6B). In *F. bucharica* grown at 18°C, the lack of BCs was consistent with high *FbGA20ox4* expression level in both SDs and LDs, and only flowering forced the topmost AXB to continue the growth of the leaf rosette sympodially. At 11°C, however, BCs were absent regardless of the downregulation of *FbGA20ox4* and the cessation of stolon formation. These findings suggest that BC development in *F. bucharica* was inhibited by factor(s) other than apical dominance, even after the downregulation of *FbGA20ox4*. This contrasts with findings in a stolonless *ga20ox4* mutant of *F. vesca* that exhibits strong apical dominance and forms BCs only after flower induction or decapitation of the shoot tip (Andrés et al., 2021). Also, columnar apple trees show remarkably strong apical dominance, and almost all of their axillary shoots develop into reproductive short shoots (Kelsey and Brown, 1992), analogous to *Fragaria* BCs. These trees have ectopic *MdDOX-Co* expression in shoots, in addition to its normal root-specific expression pattern, which hampers GA biosynthesis and leads to columnar phenotype (Okada et al., 2020; Watanabe et al., 2021).

Striking differences in BC development of *F. bucharica* and *F. nilgerrensis* may be adaptations to their native habitats. *Fragaria bucharica* is found at high altitude regions with very short growing season in Himalayas (Hummer et al., 2011; Johnson et al., 2014). Such alpine areas are usually dominated by species with low reproductive vigor that favor vegetative propagation over the comparatively riskier sexual reproduction (Billings and Mooney, 1968; Klimeš et al., 1997; Grime, 2001; Zhang et al., 2010). Therefore, the lack of branch crowns that limits the number of inflorescences to a maximum of one per plant may be an adaptive trait that makes vegetative reproduction through stolons a primary reproductive mode in *F. bucharica*. *Fragaria nilgerrensis*, in contrast, is native to habitats with very mild winters and long growing seasons in the Southeast Asia. Such conditions are more suitable for sexual reproduction, which may explain why this species develops abundant BCs in SDs in autumn/winter to enable the plant to form several inflorescences during the following growing season.

## Conclusion

In this work, we provide the first phenotype and gene expression level analyses on the control of flowering and axillary meristem fates in several wild diploid *Fragaria* species under controlled environmental conditions. We show that the examined species feature a wide range of flowering responses, and variation in *TFL1* regulation is the key to understanding the different responses. Moreover, we show that the environmental regulation of *GA20ox4* varies in the three studied species, and this variation is associated with differences in stolon development. Finally, our results on BC development suggest diverged regulation of this process in *F. vesca*, *F. bucharica*, and *F. nilgerrensis*. To summarize, the diverse phenotypical responses (Supplementary Figure 3) provide an excellent starting point for carrying out further experiments to elucidate the genetic bases of these responses. We anticipate that such studies would provide new means to control yield formation in Rosaceous fruit and berry crops through (1) altered flowering characteristics based on modifications of *TFL1* regulation, and (2) improved plant architectures by optimizing the balance between the formation of short and long shoots.

## Data Availability Statement

The original contributions presented in the study are included in the article/**Supplementary Material**, further inquiries can be directed to the corresponding author.

## Author Contributions

GF, JA, EK, and TH designed the experiments. KO provided diploid *Fragaria* species plant materials and participated in the selection of genotypes for the study. GF and JA carried out the experimental work and statistical analyses and drafted the manuscript. EK and TH supervised the work and edited the manuscript. All authors commented on and accepted the final manuscript version.

## Funding

The research was funded by China Scholarship Council (Scholarship no. 201706510014 to GF) and Academy of Finland (grant no. 317306 to TH).

## Conflict of Interest

TH was employed by company NIAB EMR.

The remaining authors declare that the research was conducted in the absence of any commercial or financial relationships that could be construed as a potential conflict of interest.

## Publisher’s Note

All claims expressed in this article are solely those of the authors and do not necessarily represent those of their affiliated organizations, or those of the publisher, the editors and the reviewers. Any product that may be evaluated in this article, or claim that may be made by its manufacturer, is not guaranteed or endorsed by the publisher.

## Footnotes

^1^ www.hansabred.org
