# Supplementary material for: Natural Variation in the Control of Flowering and Shoot Architecture in Diploid Fragaria Species
Source: Front Plant Sci. 2022 Feb 24;13:832795. doi: 10.3389/fpls.2022.832795 (PMC8926021; doi:10.3389/fpls.2022.832795)
Supplement: Supplementary file 1 [file Data_Sheet_1.docx]

Supplementary Material

*F. vesca*_*TFL1*  TTAGTTTTATTTCACGAATTCAATTACTAGGATTCCTAATAATTATATTACTTTTTAGTCTTATAAGACAAACTTCTATATATCCTTGAGAGTGATGATCATAAACTTAAA-CTTTGCATATCATTTTCAAGTTTCAGAGCAAACTAGTAATAATATTAACTGACGTTACCTTACTAATAACTGATATATAAGAGCATCT 199

*F. nilgerrensis_TFL1* -----------------------------------------------------------------------------------------------------------CAAATGTTTTTAT-T-ATTT--AA-TTT--G--CAAATTTGTT-T--T-TGAAAT-AC-TTA--TGCCGAATG-C--ATTTTTA-GAG--T-T 69

*F. vesca*_*TFL1* TCAACATCTTTCATATTTTCTTAAACTTCTCAATTTTAGAGATTTTG-ACTTTTGTTTCTCTATTATGAAGAAATTTAAAAAAAAAACGGTTAGAGATGC--TCTAATAA-TTCA-TTCAGAGAATACCAGTGTATTTCCATAAG--CTTCGA-GACTC-CACAG--CTTGAAAAT-ATTGGAAGAGACAAGGC-C--G- 383

*F.* *nilgerrensis_TFL1* TGAAAAT-TTGCA-ATT--C--AGTCT-CTTA-TTTTC-A--TTTCGAAAAATTGTAT-T--AC-A-GAAGTTGTTTTATCAATCACCTGTTC-A-ATTCAGTCTCTTAATTTCATTTC-GA-AA-A--ATTGTATTAC-AGAAGTTGTTTTATCAGTCAC-CTGTTTTTCCGATTCAA-GAAAAAGA-AATTCACTTGT 242

*F. vesca*_*TFL1* GG-AC-ATAATTTATAT---AT-AGATTCGATTCGGGGA-GCA--A-TAGAG-AGAAAAGAA--CC-GTGAGGA-GGA-AAAGGA-GGAAT-GAAACTACT-TAAAAATATAAAGAGCGAAAAGAAATAAAGGGATACGTTGA-AACATTGGGCATGGTGAAT-GCAGAGAGTTGCAGAAGAGATACAGCTAGCTTCGGC 562

*F.* *nilgerrensis_TFL1* GGCACGTTTACTTACATGGAATAAAATT-GAAAAGTGAATGAATGATTCCAGCAGAGGAGAATTCCAGCGTTTACTTACACATAATGGAATCGGAATGAGTGTGGG--TCTCACCTCCTAATA-AAG-AATCG-ATTCCTTGACAACCTTGA--ATTGT-AATACCAAAGGGG-G-AGATGAGATT-A--T-GAAT-G-- 424

*F. vesca*_*TFL1* ATATGCAGCAGAGAAAAGTACTTTGGCTTCTTGTTATAGCGAATCCACTCCTTGATATCTAGTGCTTAATTGCCAATTCTGGAACACTTCTCTGCAACACT--TTA-TTCACTTTGGCATGGGTTGTA-GCACTCGTCTGAGATGACCTCTCCATCAGCACTATCAGTGG--GTGGGTTCCCTTTTGCCTTGTGCTTCTG 756

*F.* *nilgerrensis_TFL1* ATAC-CATCAG-GAAT-GAACTTTTA-TTCCGAAAATACCCACTTTAAT--TT--TAT-TA-TGCT-AATAAT-A-TTGAGGAAT--TTGTTTAAGATGCTAATAATTTCAATTTTTTTTC--TTAAAGGCTGTTTTTT-ATATG-CTAATA-AT-AT-ACTATCTGTGGAAGTTGTTTAATTTTTT--TTAAATTTATT 600

*F. vesca*_*TFL1* TCTCTCGTTGCTGA-AAGAAGTCTTTGAAT-TACCA-AAATAACCAAG-AGAATCTCTCAAAATG-GTAAATTCCCATATGAGTATACTTGCTTGTAAATGAGAAAGGTTCTGTGGTGCA-ACACATTC-AGCA-AATTTTCTTATTCAGTTTTCAGTATACCCTATCTGGAA-AAGATGAAGGTTTTCTGTTACTAGCT 947

*F.* *nilgerrensis_TFL1* TATAAAATTGCTTACTTGA--T-TTA-AATATAATATTATTAAA-ATGTATAAACG-T-ATGATGTCTTAATTTTG-T-TGTTCA-ACC--CTTACAACTT-GC--GGTT-TGAGATACACCCAAAATCTAGTAGTAGGTCCAAATG-A-TTTTAA-TAAATC--AT--GGAAGAAGA-GA-G-TTTA-TGCAAGGA-C- 771

*F. vesca*_*TFL1* GTATGAATCTTCAGGA-AGAACAGAGAATCCGAGACATTTTTAATTTGCTGT-TTCGTTATAAAATAGCATG-AAGTGACAGCTAATT-CAAGGACCCGGCCCACATGCAT-GATGGATTGTTTTTTTTTTTTTTTTTTC-T--TTTATGGCAA-AGA-ACTTTTAAGAAGA-ACAC-ACTGTATTTTTTTTTTTTGAAG 1135

*F.* *nilgerrensis_TFL1* G-A-GA-T-TGCAATATACA-CA-AGCA-C--ACACGT----AGTCT-CTGTATTA-T-ATTAA-TAATATGTAACC-ACA--TGATTACAATCAATA--C--A-ATGAATAGAAATACTCTTTGGTTTGTTGATTATACATAACTTAT--CAACTGAGACAATCAAAACGACAAACAAAACTAGATCAACATATA-AGG 943

*F. vesca*_*TFL1* AAGAATAATTCAGCAGGTTTACAGCCAATCAAGAACTGATTCCAAAGACCATGCATATTA-GAGTACGTTTGGCACATT-AGTATAGAATT-CTTATTTTTATAAAATAATGTATCATGTATTAAATATAA----AAATTTGAATAGAATATCTTGTATTTTTTTTGTTTATATGTTTCTCTAGCCACAAAATTCACTTG 1328

*F.* *nilgerrensis_TFL1* AA-AA-AAT--A--ATGTTGAGAGTGA-TCGA-AA-TGTGT--A--GTTTATTTTT-TCATGGGAA-GTTTAGTA-ATTAAATA-ATAATTAATTACATTCATGTTCTAAT-TC-CATGTGGTAAGTAAAATGCTCAATG-GAATTGAA-ATCTCC-ACATACTAT-TCCAT-TCCGTCTCT--C-A-----TTC-CTTA 1110

*F. vesca*_*TFL1* T-TCTAGC-TATCAATAAATCACCTGTTTA-TCCGATTCAAGAAAAAGAAATTCACTTGTTTCTTAC-GATTCCTTAAAATCTTAAATTGATGCAAGGTTGAAAAGAGTGTAATAGGTTATTGCACTCTGTCATTTTTTTTTATCAGGTAAATTACAACTGTGTGAGTCGAACTCATAAC-CTCTCACTTACCAAAA-AG 1522

*F.* *nilgerrensis_TFL1* TGTCTTTCAT-TCC-TGA-TGAA-T-TTCATTCCAAGT-AAGTAAACGCG-T-CA-TTGTTTCTTACAG-TTCCTTAAAATCTTAAATTGATGAAAGG---A---G--TGTAATAGGCTATTGCACTCCGTCATTTTTTTT-ATCAGATAAATTACAACTGCGTAAGTCGAACTCACAACTTTCT-ACTTACCAAAAGAA 1290

*F. vesca*_*TFL1* AG-GAGAGACTATGTTACTAGACTAAATGGCACTTCATCATCTTTGTCTGTTAATTTATTTGTTGAATATCCAACATCTTAGTTTTCCTGCAAGGCATTTCCTATGTAATTAAATTCCAAGTATATATGACATCTTATCATTCCTTAAGCCTGATCATTTCACA-G-----GTCTCCCAAAACTTACCTCCTTCATCTTT 1715

*F.* *nilgerrensis_TFL1* AGAG-GAGACTATGTCACTAGACCAAATGACACTCCGTCATCTTTGTCTGTTAATTTATTTGTTGAATATCCAACATCTTAGTTTTCCTGCGGGGCATTTCCTATGTAATTAAATTCCAAGCAT----GACATCTTATCATTCCTTAAGCCTGATCATTTCACACGTACATGTCACCCAAAACTTACCTCCTTCATCTTT 1485

*F. vesca*_*TFL1* CATTGTATGAGTGAAAACTTCCCCTCGGCAAAGCCATTATCGCCAAAACTTCTTTTGAGTCTCAAGTCTTAACCATTCTACATTCATGCAAGAGTACGTCAACATGCATGATCGACGGTTGAAATTGATTCACAGTTTCGGGTAGTACATCTTGTCCAGAAAGAAGAGAACATTTTCCTCAAGAAAAAGCATTAAACCCT 1915

*F.* *nilgerrensis_TFL1* CATTGTATGAGTGAAAACTTCCCCTCGGCAAAGCCATTATCGCCAAAACTTATTTTGAGTCTCAAGTCTTAACCATTCTACATTCATGCAAGAGTACGTCAACATGCATGATCGACGGTTGAAATTGATTCACAGTTTCGGGTAGTACATCTTGTCCCGAAAGAAGAGAACATTTTCCTCAAGAAAAAGCATTAAACCCT 1685

*F. vesca*_*TFL1* TTGCATTGATGTGACATGCACAGAGAGAAAGAGAGGACGGGAGGTGCGAGAGACTAGCTAACAATGTTTTAAACTTCTAAATTTCTTCTAATGTCCCATGATATTCTATTCATTTATACTCTTCTTCTTCTTCTTCTCTCCCAAAACACTTGTGAGATCTAGAGCAAACTAGTTAGCGTACTGCAGATCACTATAAATAT 2115

*F.* *nilgerrensis_TFL1* TTGCATTGATGTGACAAGCACAGAGAGAAAGAGAAGACGGGAGGTGCGAGAGACTAGCTAACATTGTTTTAAACTTCTAAATTTCTTCTAATGTCCCATGATATTCTATTCATTTATACTCTT-TTCT-CT-C--C-C----AAAATACTTGTGAGATCTAAAGCAA-CTAGTTAGCGTACTGCAGATCACTATAAATAT 1874

*F. vesca*_*TFL1* AGACCAATACAGAGATTCACTTGCAAGCAAAGGCAAATATTAGAAGAGCTAATCCTTTCCCCGAGTTTATCCATCTTTGATTTATCTGTACAACCTTTTCTCTTCTCCCTCTTTCGAGTTCTAACA**ATGGCAAGAATGTCGGAACCTCTAGCTGTTGGAAGAGTCATAGGAGATGTTCTTGATTCCTTCACCCCCACTAC** 2315

*F.* *nilgerrensis_TFL1* AGACCAATACAGGGATTCACTTGCAAGCAAAGGCAAATATTAGAAGTGCTAATCCTTTCCCCGAGTTTATCCATCTTTGATTTATCTGTACAACCTTTTCTCTTCTCCCTCTTTTGAGTTCTAACA**ATGGCAAGAATGTCGGAACCTCTAGCTGTTGGAAGAGTCATAGGAGATGTTCTTGATTCCTTCACCCCCACTAC** 2074

*F. vesca*_*TFL1* **AAAAATGATTGTCTCTTACAACAGCAAGCTCGTCTGCAATGGACATGAGCTCTTCCCTTCTGCAGTCACCGCCAAACCTAGAGTTGAGATTCAAGGAGGCGACATGAGATCATTCTTCACTCT**GG----TATACATCATTTTCTTTCTTTCCTCCTTTTTCCGCCTTTTG-TTTTATATCAGATCTACCAGATCGCTAAC 2508

*F.* *nilgerrensis_TFL1* **AAAAATGATTGTCACTTACAACATCAAGCTCGTCTGCAATGGACATGAGCTCTTCCCTTCTGCAGTCACCGCCAAACCTAGAGTTGAGATTCAAGGAGGCGACATGAGATCATTCTTCACTCT**GGTATATATACATCATTTTCTTTCTGGCCTCCTTTTTCCGCCGTTTGTTTTTTTATCAGATCTACCAGATCGCTAAC 2274

*F. vesca*_*TFL1* TTTAGTTATTTTCACA**GGTAATGACAGACCCAGATGTTCCTGGCCCTAGTGATCCTTATTTGAAAGAGCACCTGCACT**GGTATATATCTTACAGTTAATACTG--TATAT-T-ATCAAACCTAGAAACGAAGAAACAAAGACAATAACAATTCAGAT-A--ATTAATGACTTCAATGTTCAAACTAGATATATATGTTGT 2701

*F.* *nilgerrensis_TFL1* TTTAGTTATTTTCACA**GGTAATGACAGACCCAGATGTTCCTGGCCCTAGTGATCCTTATTTGAAAGAGCACCTGCACT**GGTATAT--CTTACAGTTAATACTGTCTATATATCATCAAACCTAGAAACGAAGAAACAAAGACAATAACTATTCAGATAATTATTAATAACTTCAATGTTCAAACTAGATATATATGTTGT 2472

*F. vesca*_*TFL1* CATATTCTAGAAATTTGCAGTATTGACCAAACAAATTGTCCAGGTTAATTTATAGATCAATTGCACTCCTGCTTGTAATTTCCAGATTCAAAGAAAAGATTAGTTATATTAATTATTGTTAATTTATTCTTTCTCCTTTACATGGTCATTCACGTATATATTCAGAATTGTAATCAGAAAATCTTTGGATCTGGAAATAA 2901

*F.* *nilgerrensis_TFL1* CATATTCTAGAAATTTGCAGTATTGACCGAACAAATTGTCCAGGTTAATTTATAGATCAATTGCGCTCCTGCTTGTAATTTCCAGATTCAAAGAAAAGATTAGTTATATTAATTATTGTTAATTTATTCTTTCTCCTTTACATGGTCATTCACGTAT-T---CAGAATTGTAATCAGAAAATCTTTGGATCTGGACATAA 2668

*F. vesca*_*TFL1* AAGCAAGTAAAAATTACATATTATGTCACGAACTCTTAACTAGAAAAACTAGCAAGGTATAATTGTGGAGTATATACATGCCAATAAGCTTGGATCTAACAGTTTTGCCATGAAATTGAACTCTACA**GGATTGTGACAGACATTCCTGGCACCACAGATGCTACATTT**GGTAGGTTAATTCAAACTAGTTCATGAGCTAC 3101

*F.* *nilgerrensis_TFL1* AAGCAAGTAAAAATTACATATTATGTCACGAACTCTTAACTAGAAAAACTAGCAAGTTATAATTGTGGAGTATATACATGCCAATAAGCTTGGATCTAACATTTTTGCCTTGAAATTGAACTATACA**GGATTGTGACAGACATTCCTGGCACCACAGATGCTACATTT**GGTAGGTTAATTCACACTAGTTCATGAGCTAC 2868

*F. vesca*_*TFL1* CAGTACGTTATACAGCTAGTGATGATCAGCACTGATATTGTGTATATACTACATTAATTAACA**GGAAGAGAAGTGGTGAGCTACGAGATGCCAAGGCCAAACATAGGCATCCACAGGTTTGTGTTTGTTCTCTTCATGCAAAAACGAAGGCAGTCGGTGAACCCGCCTTCCTCAAGGGATCACTTCAACACCCGAACCTT** 3301

*F.* *nilgerrensis_TFL1* CAGTACGTTATACAGCTCGTGATGATCAGCACTGATATTGTGTATATACTACATTAATTTACA**GGAAGAGAAGTGGTGAGCTACGAGATGCCAAGGCCAAACATAGGCATCCACAGGTTTGTGTTTGTTCTCTTCATGCAAAAACGAAGGCAGTCGGTGAACCCGCCTTCCTCAAGGGATCACTTCAACACCCGAACCTT** 3068

*F. vesca*_*TFL1* **CGCAGCCGAAAACGACCTTGGTGTTCCTGTTGCTGCCGTTTACTTCAATGCACAGAGAGAAACGGCAGCAAGAAGACGCTAG**CTAGCTAGGCACCTTGCAGGGAGGCAGGCCAGATCCCAAAAGATCTCTTGCTATAACCA-GAATTAATCCTCCCTAATTAAATAAAAGAGAAGTGTGTAGTAATGAATTAATCCTCCC 3500

*F.* *nilgerrensis_TFL1* **CGCAGCCGAAAACGACCTTGGTGTTCCTGTTGCTGCCGTTTACTTCAATGCACAGAGAGAAACGGCAGCAAGAAGACGCTAG**CTAGCTAGACACCTTGCAGGGAGGCAGGCCAGATCCCAAAAGATCTCTTGCTATAACCATGAATTA--CCT----AATTAAATAAAAGAGAAGTGTGTAGTAATGAATTAATCCTCCC 3262

*F. vesca*_*TFL1* TTTGGCTAGGTTATTAGGCTAAAATTACTCGCATCTTACCAGAATAATTTTCCCAAACGGAGCTGTACGTGAGTGTTTGTGTGACTGTCATTAGCTTTTCTTTT-TC-CTCTCTCCGGCCTGGAATGTTATGAAGTAAATTTGTTCTTAGTTAATTTGATGTACCGGTAAAGTATCTATTCAATATAGATACATGTCTTT 3698

*F.* *nilgerrensis_TFL1* TTTGGCTAGGTTATTAGGCTAAAATTACTCGCATCTTACCAGAATAATTTTCCCAAACGGAGCTGTACGTGAGTGTTTGTGTGACTGTCATTAGCTTTTCTTTTCCCTCTCTCTCCGGCCTGGAATGTTATGAAGTAAATTTGTTCTTAGTTAATTTGATGTCCCGGTAAATTATCTATTCAATATAGATACAAGTCTTT 3462

*F. vesca*_*TFL1* GGTTTTGAATCATCGTTGATCACTGGAAATCTATAAAGGAACACGTGGAACAAACCCATTATCTG------TC---T---ATTTCAATTCTCTTCTTCTGGTTAAGGTTTAGGGCATCCATTTCATTTATG----ATGCAATTAG-AA-TCTGTGTTCAAGCTTTGTCCACCCATGGAGAAAAATTCAACCTAGGTTG-- 3878

*F.* *nilgerrensis_TFL1* GGTTTTGAATCATCGTTGATCACTGGAAATCTATAAAGGAACACGCGGAACAAACCCATTATCTGTCTATTTCAATTCAAATTTCAATTCTCTTCTTCTGGTTAAGGTTTAGGGCATCCATTTCATTTCTGAAAAAT-CGATCAGCAACTCAATCAGAAAGGATCGGCAGTTC--GGC-ACCAAT--AA--TAT-TTGCA 3653

*F. vesca*_*TFL1* A-ATGCCACA-TACAAATC-TCACCCAAGTGAAAAAAAACCCACTTTCAAC-CCATAA-AATGGGCT--AT--CTCATTTTCCAACCTAGGTTGGATTGT-TGGGAGAGAAGTGACCCA-G-GTTGGATGA-GAAATCCAGCCCAGGTTGC-TTTGTGACA-CCTCAACCGAGCCCA-CTTGCCCATTTTTC-ACGTGGT 4061

*F.* *nilgerrensis_TFL1* AGATGGCTCATTTTTTTTCGTCATTCGGG-GAATCAA--CTAAGTTGCCTCTCCAAAACAAAGT-CTGAATGGCTGAAAATA-AACCTACAACAAAA-GTCCGATAAACAAGC-ACAAACGAACTA-ATAACAAAAAGAAACTTAATTTCCACTTGAGAAATTAT-ATTC-A-CATATCTTTA--ATTTATCGATACACA 3840

*F. vesca*_*TFL1* GTGC-ATCATCTTCTTG-CC---TCCCACAAACAAACGCGCTTGGAGTTG--GC-GT-TTTGCGCACGAACCAACAACAGCCATGCAATAGCGCGTGTATTGTGCCATTTTGT-CGAGACATACTGCGAGCTTCCTAACCCCCCCCCCCCCCCCCCCCCGACACGTGTCACGTCACCGA-TGTTGCTAAC-TTTCATTTT 4249

*F.* *nilgerrensis_TFL1* CCCCTAAAATATTTTTGTCCAATTTTCACTCA-ATTTTC-CTTT-A-TTGCCCCAATGTTTGAAAACATAGCAA-AACA-C--TGTAATTTTTC-TGGAAAATAA-ACTGCATACTA-A-ATAAAGA-AT-TAAAGATGGGAAGACCAAACCTATTCGCAAAACC---CAC-TC-CCAATTTTTTC-AACATTT--TTTT 4018

*F. vesca*_*TFL1* GAATG-TAAC-GGTCA-AAAGATCTGAGCCGTTGCTGGAGCAATCTGACTGTT-GTA-ATTAGCTAGCTTTTTTTTTTTGAATCTGACC-GTTTTGGAATATTTTCAACGATGAGAAAAAAGTT-TATAACGGTAA-GAAAAAAAAT-ATCCCCT-AT-AACGGGAAGAAAAAAATATTAAAAAAATTTGTATAAT-CCC 4437

*F.* *nilgerrensis_TFL1* -ATTGCTACCAGGTAATCAATTTTT-ATTCGT-G--GGTG---T-TGG-TGTTGGTAGATTTGG-AGTAAGATTCTTTTG--T-TGACCAATTCGGGAA-AT---CAAC--T-A-ATTAA-GTTGCCTCTCCAAAACGGACTGAGATCCTCTCCTCATAAACCTCT-GCTAATATT-TCCTAATAAGT-GTATTCTGACC 4192

*F. vesca*_*TFL1* CTATAAT-TAAAT--AC-CCATCACA-AT-TT-T-AC--ACATCCGCACCAATTTCTC-TAAG-ACGTCAAAATTTC-CTAGTC--TCCAAATTC-CGT-AGCTTTCATCTTCTAGGTTTTACC-TTAT-TTC-AATCATTCTCT-CT---CTT-CT-CATTCGCACTCTTCACAATG-AATAATTTGTGGCAAAGGATC 4610

*F.* *nilgerrensis_TFL1* GTTTGATCTTAATGAACGGC-TAAGATGTCTTCTCTCTAACTTAAAAGTTATTTTCTCATATGTTCTTCTAT-TCTCTCTC-TCAATC-AAATTCTCTTCCGATGT-ATTTTCTCG-TC--ACCGTCATCTCCAAACCCTTCTCTCCTCGCCTTACTCCAAAATCAC-CTTC-CACCGCCATCACC-GT--CAACGCCGC 4379

*F. vesca*_*TFL1* CG-AAAATCTCAAGAAGAAGACGACTAAGATGATCTAGCAA-CTA-ACAAT-A-TCGT-TATCGCAGC--CATTGCTCACCTTGAAGCTGAAAACCAACCTCGAG-GTCGCGGAT-C-TCGTCCAGGTCGTCGTCCAAAC--CAACCTGGAGAAAGGGAGGACAAAGGCAAAGGT-ATGCT--TGAAGATT--AT-TTTG 4791

*F.* *nilgerrensis_TFL1* CGCACCACCACAAACACAA-AC-AGTAACA--ATC-A-CAATATATTCCATGATTAATCAAACCCAGCTTCTTT--TCACCAA-AAGAAAAAATCAAACC-C-AGCTTCCAGAATCCATATTACAAGTC-TC--C-AAACTTTAA--TGAA-ATTGCGAAGT-AAATTAAAAGTTAAAGCTAACCCA-ATTCAATATAT- 4558

*F. vesca*_*TFL1* TCGAACG-TTCTATT-T-TTAATGATGA-GGA-ATTCC--GAGTTCGCTACAGAATGAGC--CACGGTGTCTTCAACCGCATATGTGGTGACCTTTGCAACTACGAT-CGATATTTTGTTC---AAA--A--AA-TCGATGC---TTCC-AA-GAAGGTCGGTCTACTTCCCCA-ACAAAAGA-TGACATCCTCTTTACA 4966

*F.* *nilgerrensis_TFL1* TCCA-CGATTA-ATTGTATCAAACAAAATATATATTCCATGA-TTAA-T-CAAACCCAGCTTC-CAGA-T-TACAA--GAA-ATCTAATTATCTAAGCGA-T-C-ATCCGAAATTATATTCATGAAATTATGAAGTTGAAGCAAAACCCAAATGAAA-TCGAACAAAA-CCCAATCCAATAGACCGAAAGATTCATG--- 4739

*F. vesca*_*TFL1* GATGCTCGCCTACGGCACA-TCCGCA--GATCAATGTGCTG-AGT-ATTGTC-A-G---ATGAC-A--AAATCTACCT--CTATCGAGTGCCTCCAACGATTTACAAGAGGAAT-TGTTGCTCTTTA--C-TCAGC--A-GATTACCTTCAAGCTC-TTAATCCAGTCGATCTAAAAAAACTTCTTGC-TA-AAGGTGAA 5141

*F.* *nilgerrensis_TFL1* GTTGGTGGC-TACGATAGAGGCGGTAGGGAGGAG-GAGTTGAAGTGATCGGCGACGACAATGGCGAGGAAGTCTTGCTGGGT-TCGGGAGAATG-ACCGGTGGAGA-GA--AATGGGTTGGT-TTGAGGCGGCAGCGGAGGAGGGGAGTAAGGGTCGAAAATATAGTGGCTGAGAAGGGGGTGATGGCTTAGGAGGGGGA 4931

*F. vesca*_*TFL1* AAGCGAGGT-TTTCCAGGGATGATTGTGAGC-AT-CGACTGCATGCAT-TGGCAATG-GA-AGAATTGTCCATCCG-GTA-ATTGGGCT-GGTGAATA--T--AGTGGTAGA---AAACACATCCCCA-CTATCATTCTTAA-AG-CGGTC--GCATTATACGA-CACTTGGATATGA-CACAC-CTTCTTTGGA-A-TA 5315

*F.* *nilgerrensis_TFL1* GAG-GATTTGAGACTTGAGA-GAT-GTAATCTCTCCGAC-G-A-GAATATGGGAGGGAGAGAGAAA-GAG-ATTGGAGAAGATAGATATGGGAAAATATCTGAAGGGGTAGACTTAGAGACATCTTGACCTTTGATTAAGAATGGATGGCCAAG-ATTTTCTGATGAGGAA-ATATTAGCAGACAAATATGAGGAGAGGA 5121

*F. vesca*_*TFL1* CCCGGGT-CATGAAATAACCTCAATGTCCTAGCAAAATCCTCGTTGTTTGACGAGCTTACTATCGGTCAAGCACCTAAGATCCAGTTCCAAGTGAATAACAGAGTCCACAAGTTGGGCTACTATATATCTTGCCGACAGTATATATCCAAAGTGGGCGACTTTTGTAAAACTATTCCACATCCCACCAGTCCTAAGGATA 5514

*F.* *nilgerrensis_TFL1* TCCGGATCCCTCCAA-AACA--AA-GTC-T-G-AAT-------------------------------------------------------------------------------------------------------------------------------------------------------------------- 5150

*F. vesca*_*TFL1* TTACATTTTCCAAGGCCCAAGAGGGGTATAAGAAGGATGTGGAGAGGTGTTTTGGTATTTTTTGGAGTCACATTTTGGTATTATTAGAGGAGC 5607

*F.* *nilgerrensis_TFL1* --------------------------------------------------------------------------------------------- 5150

**Supplementary Figure 1**. DNA alignments of *TFL1* from *F. vesca* and *F. nilgerrensis*. Both sequences contain *TFL1* exons (marked in red font) and introns, and putative promoter sequences 2000 bps upstream of the transcription start site and 2000 bps downstream of the stop codon. Conserved base pairs are marked by similar background colors. Sequences were retrieved from the *F. vesca* v4 and *F. nilgerrensis* YNU v1.0 genomes both available at the Genome Database for Rosaceae ([www.rosaceae.org](http://www.rosaceae.org)). Sequence alignments were generated using MAFFT version 7 (<https://mafft.cbrc.jp/alignment/server/>) and visualized using the Color Align Properties tool available at Sequence Manipulation Suite version 2 (<https://www.bioinformatics.org/sms2/index.html>). Note: the *F. vesca* v4 reference genome is from an everbearing ‘Hawaii-4’ accession, which harbors a well-characterized 2-bp deletion within the first exon of *TFL1*. The FIN56 accession used in our studies does not have this deletion (Koskela et al., 2012) and therefore the deletion was replaced by FIN56 sequence in this alignment. The bases are marked by blue background.


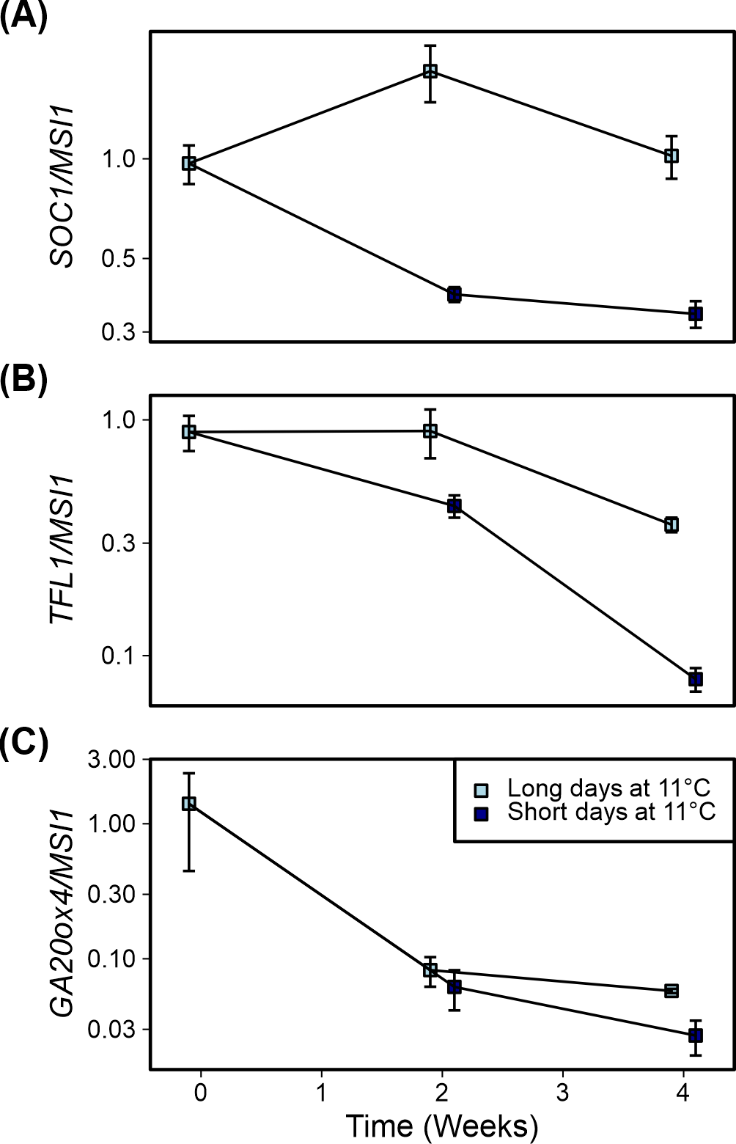


**Supplementary Figure 2.** Gene expression patterns in *F. vesca* grown under SDs or LDs at 11°C. *SOC1* (A), *TFL1* (B) and *GA20ox4* expression (C) in shoot apical meristem samples. 5-weeks-old seed propagated plants were grown in LDs (18-h) or SDs (12-h) at 11°C, and shoot apical samples were collected at the beginning of the treatments, and 2 and 4 weeks later. Week 0 *F. vesca* samples were used as calibrator for relative expression analysis. Error bars represent the standard error of the mean (n = 3-4).


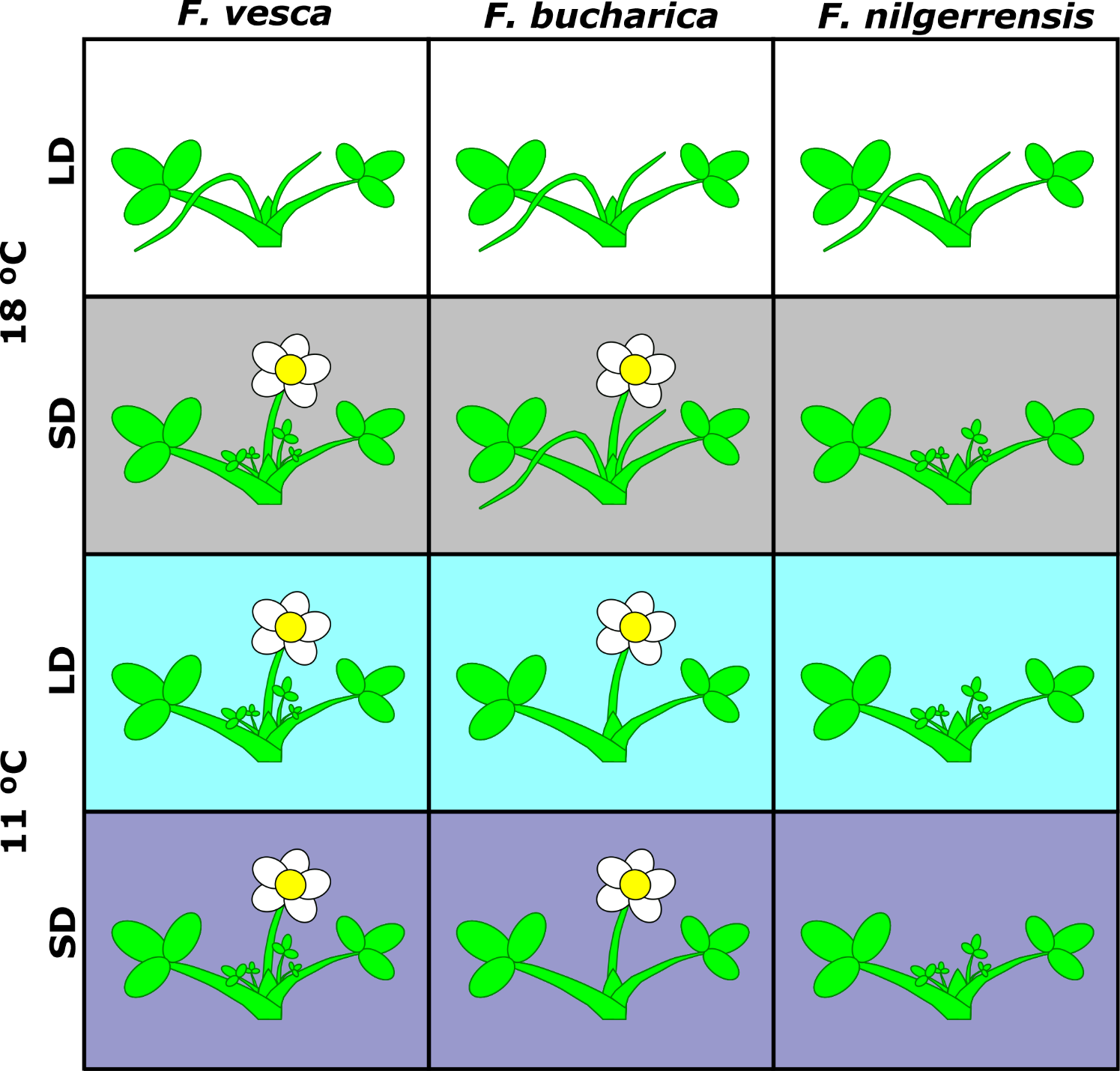


**Supplementary Figure 3**. Phenotypical responses of *F. vesca, F. bucharica* and *F. nilgerrensis* to different environmental conditions. The cartoon depicts the typical responses observed in the three species. In *F. vesca*, cool temperature (11°C) or SDs at 18°C promote both flowering and branch crown development, whereas LDs at 18°C promote stolon development. In *F. bucharica*, flowering is advanced by the same environmental conditions as in *F. vesca*, although branch crown development was not observed under any tested condition. In *F. bucharica*, a temperature of 18°C promotes stolon development independently of photoperiod. None of the tested conditions unequivocally advanced flowering in *F. nilgerrensis*, although branch crown development was promoted by the same environmental conditions as in *F. vesca*.

**Supplementary Table 1.** Plant materials used in this study and their origin.

| Species | Accessions | Origin |  |  |
| --- | --- | --- | --- | --- |
| *F. bucharica* | *F. bucharica* #1 | Ushu-Valley, Khyber Pakhtunkhwa, Pakistan |  |  |
|  | *F. bucharica* #2 | Geographic origin unknown |  |  |
| *F. chinensis* | *F. chinensis* #1 | Lanzhou, Gansu, China |  |  |
|  | *F. chinensis* #2 | Lüliang Mountain, Shanxi, China |  |  |
| *F. iinumae* | *F. iinumae* #1 | Honshu, Gi fu, Japan |  |  |
| *F. nilgerrensis* | *F. nilgerrensis* #1 | Leigong Mountain, Guizhou, China |  |  |
|  | *F. nilgerrensis* #2 | Yunnan, China |  |  |
| *F. nubicola* | *F. nubicola* #1 | Yadong, Tibet, China |  |  |
|  | *F. nubicola* #2 | Gori valley, Uttaranchal, India |  |  |
| *F. pentaphylla* | *F. pentaphylla* #1 | Chengdu, Sichuan, China |  |  |
|  | *F. pentaphylla* #2 | Gyala Peri, Tibet, China |  |  |
| *F. viridis* | *F. viridis* #1 | Kuscherla, Altai Republic, Russia |  |  |
|  | *F. viridis* #2 | Meissen, Saxony, Germany |  |  |
|  | *F. viridis* #3 | Tianshan Mountain, Xinjiang, China |  |  |

**Supplementary Table 2.** RT-qPCR primers used in this study.

| Gene | Forward primer sequence | Reverse primer sequence |
| --- | --- | --- |
| *FvMSI1* | TCCCCACACCTTTGATTGCCA | ACACCATCAGTCTCCTGCCAAG |
| *FvSOC1* | ACTTGCTGGGTTCATTTTCC | GAGCTTTCCTCTGGGAGAGA |
| *FvTFL1* | CTGGCACCACAGATGCTACA | AACGGCAGCAACAGGAAC |
| *FvGA20ox4* | CCAGAGGAACTTGTTACTGAAGTAGG | TCAATTGACTGATTTGGATTCAGACTTG |

**Supplementary Table 3.** Number of flowered plants after long-term cool temperature treatment at 5-6ºC.

| Species/Accessions | Number of plants | Flowered plants |
| --- | --- | --- |
| *F. bucharica* #1 | 7 | 6 |
| *F. bucharica* #2 | 4 | 4 |
| *F. chinensis* #1 | 3 | 3 |
| *F. chinensis* #2 | 3 | 3 |
| *F. iinumae* | 13 | 0 |
| *F. nilgerrensis* #1 | 13 | 13 |
| *F. nilgerrensis* #2 | 3 | 2 |
| *F. nubicola* #1 | 3 | 3 |
| *F. nubicola* #2 | 10 | 0 |
| *F. pentaphylla* #1 | 14 | 14 |
| *F. pentaphylla* #2 | 15 | 15 |
| *F. viridis* #1 | 3 | 2 |
| *F. viridis* #2 | 3 | 3 |
| *F. viridis* #3 | 3 | 3 |
| *F. vesca* | 15 | 15 |
